# Improving the Accuracy and Efficiency of Respiratory Rate Measurements in Children Using Mobile Devices

**DOI:** 10.1371/journal.pone.0099266

**Published:** 2014-06-11

**Authors:** Walter Karlen, Heng Gan, Michelle Chiu, Dustin Dunsmuir, Guohai Zhou, Guy A. Dumont, J. Mark Ansermino

**Affiliations:** 1 Department of Electrical & Computer Engineering, The University of British Columbia, Vancouver, Canada; 2 Department of Anesthesiology, Pharmacology & Therapeutics, The University of British Columbia, Vancouver, Canada; 3 Faculty of Medicine, The University of British Columbia, Vancouver, Canada; 4 Department of Statistics, The University of British Columbia, Vancouver, Canada; Charité - Universitätsmedizin Berlin, Germany

## Abstract

The recommended method for measuring respiratory rate (RR) is counting breaths for 60 s using a timer. This method is not efficient in a busy clinical setting. There is an urgent need for a robust, low-cost method that can help front-line health care workers to measure RR quickly and accurately. Our aim was to develop a more efficient RR assessment method. RR was estimated by measuring the median time interval between breaths obtained from tapping on the touch screen of a mobile device. The estimation was continuously validated by measuring consistency (% deviation from the median) of each interval. Data from 30 subjects estimating RR from 10 standard videos with a mobile phone application were collected. A sensitivity analysis and an optimization experiment were performed to verify that a RR could be obtained in less than 60 s; that the accuracy improves when more taps are included into the calculation; and that accuracy improves when inconsistent taps are excluded. The sensitivity analysis showed that excluding inconsistent tapping and increasing the number of tap intervals improved the RR estimation. Efficiency (time to complete measurement) was significantly improved compared to traditional methods that require counting for 60 s. There was a trade-off between accuracy and efficiency. The most balanced optimization result provided a mean efficiency of 9.9 s and a normalized root mean square error of 5.6%, corresponding to 2.2 breaths/min at a respiratory rate of 40 breaths/min. The obtained 6-fold increase in mean efficiency combined with a clinically acceptable error makes this approach a viable solution for further clinical testing. The sensitivity analysis illustrating the trade-off between accuracy and efficiency will be a useful tool to define a target product profile for any novel RR estimation device.

## Introduction

Respiratory rate (RR) plays a fundamental role in routine clinical assessment for disease diagnosis, prognosis, and treatment in children [1]. Accurate measurement of RR is of paramount importance because an elevated RR is a marker of serious respiratory illness [2] and is the main diagnostic criterion for childhood pneumonia [1], the leading cause of death in children aged 0 to 5 years worldwide [3]. However, many studies have demonstrated clinically obtained measures to be inaccurate, lacking both reliability and reproducibility in a variety of health care settings [4]–[6].

The current recommended method for measuring RR is to count the number of breaths in one minute. This method was promoted by the World Health Organization (WHO) [7], and since 1990 facilitated by the distribution of the Acute Respiratory Infection (ARI) Timer in the developing world. The ARI Timer is a simple device providing auditory feedback in the form of ticks at 1 s intervals for 60 s. The ARI Timer has shown many limitations in the field, mostly related to usability aspects [8], [9]. The measurement duration of 60 s is perceived to be too time consuming. Also, counting the number of breaths in a fixed amount of time requires that measurements be restarted from the beginning in case of a noticed artifact (e.g. missing a breath) or distraction, further increasing total measurement time. Counting the number of breaths over 60 s can be especially difficult in sick children who may breathe at a rate that is upwards of 60–70 breaths/min. In practice, health care workers do not count RR for a whole minute, but instead count for only a fraction of 60 s and then scale up the number of breaths to 60 s. This decreases the accuracy of RR measurement by amplifying the counting error [10]. Thus, there is an urgent need for a robust, low-cost device that can help front-line health care workers to measure RR quickly and accurately. An effective way for improving efficiency and accuracy of rate estimations is incremental and continuous analysis of time intervals instead of counting events in a fixed time interval [11]. However, there is a trade-off between accuracy and efficiency which has to be addressed by design [10].

The user interfaces and the ubiquitous availability with health workers make mobile phones the ideal platforms for mobile health projects. A growing number of initiatives are leveraging the wide availability, affordability, portability, and usability of mobile phones to tackle the health challenges of developing countries [12]. The capabilities of mobile devices allow advanced rate calculations, such as time interval analysis. When such algorithms are packaged into user-friendly software applications, health workers could install the software through popular channels onto their existing devices without significant increase in cost for the health workers or the supporting health systems.

We propose a method of estimating RR in less than 60 s using a mobile phone application. RR is estimated by measuring the median time interval between breaths, and then dividing 60 s by this time interval. The time interval between breaths is measured as the user taps on the touch sensitive screen of a mobile device in time with the inhalation phase of breathing. The accurate measurement of the breath intervals using an electronic system allows for additional tests to validate the RR estimation. We use the measure of consistency (% deviation from the median tap time) to exclude aberrant breath intervals while the measurement process is ongoing. We hypothesize that:

1. RR estimations using median tap interval times can provide a RR in less than 60 s, therefore increasing efficiency compared to the recommended 60 s counting.
2. Accuracy improves when more taps are included in the calculation of the median RR.
3. Accuracy improves when inconsistent time intervals are excluded.

In this manuscript we present the development of the algorithms to calculate RR from tapping time intervals and demonstrate the gain in efficiency through experimental tests using standardized videos of children breathing at a wide range of RRs. A sensitivity analysis and optimization experiment for the novel algorithm will facilitate the definition of target product profiles and device specifications for the development of RR measuring devices that allow for shorter assessments to increase acceptance with health care practitioners.

## Methods

### Estimating RR from time intervals

RR is calculated from the median time interval 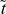 between breaths from a finite set of consecutive time intervals 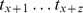, where the number of intervals in a set = z.
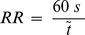
z+1 determines the minimum number of taps to complete a measurement and consequently the duration of the measurement.

### Measuring consistency of time intervals

The consistency C of a measurement is reported as the maximum percentage of absolute deviation of each time interval 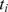 from the median time interval 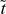 of the set:
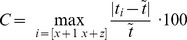



### Reporting the estimated RR

**Figure 1:**
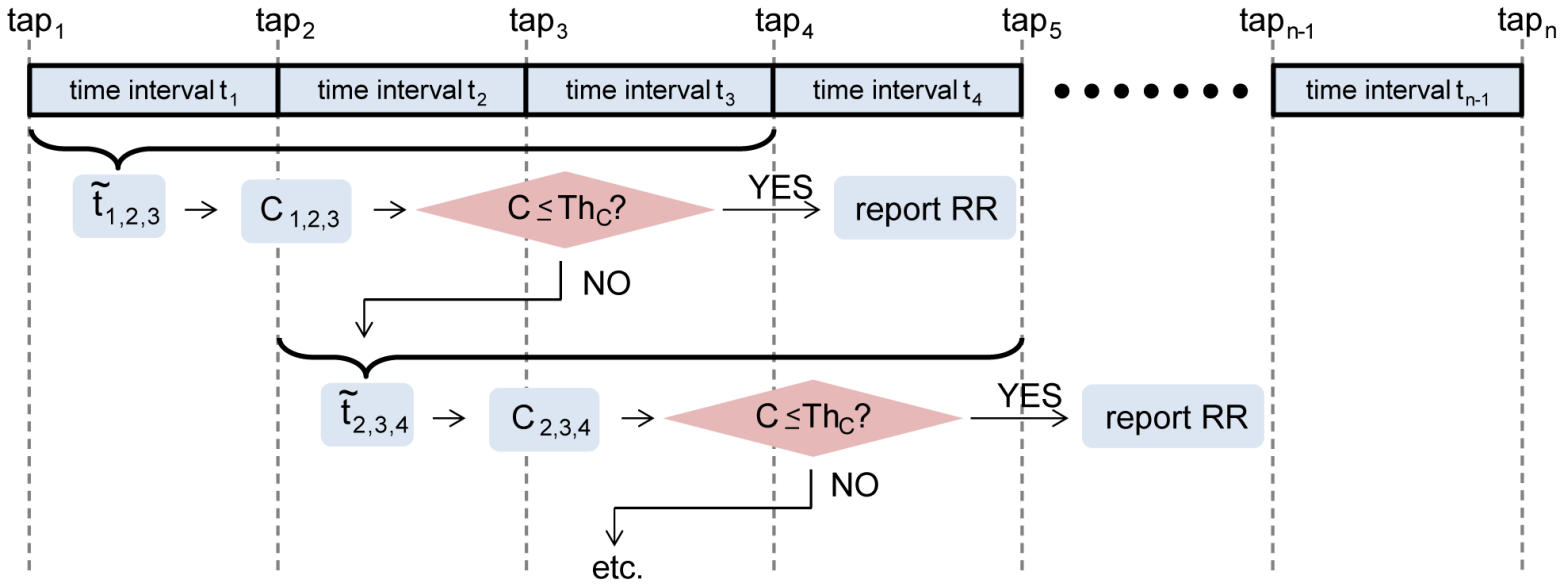
Estimation of respiratory rate (RR) with a set size of 3 tap time intervals. The median time interval 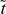 is calculated for the set and the consistency C is derived. RR is reported and the measurement stopped if C is below a specified consistency threshold 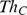, otherwise tapping continues and a new set is created, until an acceptable C is obtained.

A RR calculated from a set is reported if the consistency C is equal or lower than a consistency threshold 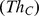. If C exceeds the consistency threshold 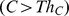, the earliest time interval is discarded from the set and a new time interval is recorded and added to the set. This is repeated until a valid set is obtained (Figure 1).

### Mobile device application

A mobile phone application called *RRate* was developed for this study. The application was built using a software framework that allows cross-platform development for medical applications [13]. A non-study version of this application is available from public software repositories for free [14], [15].

**Figure 2:**
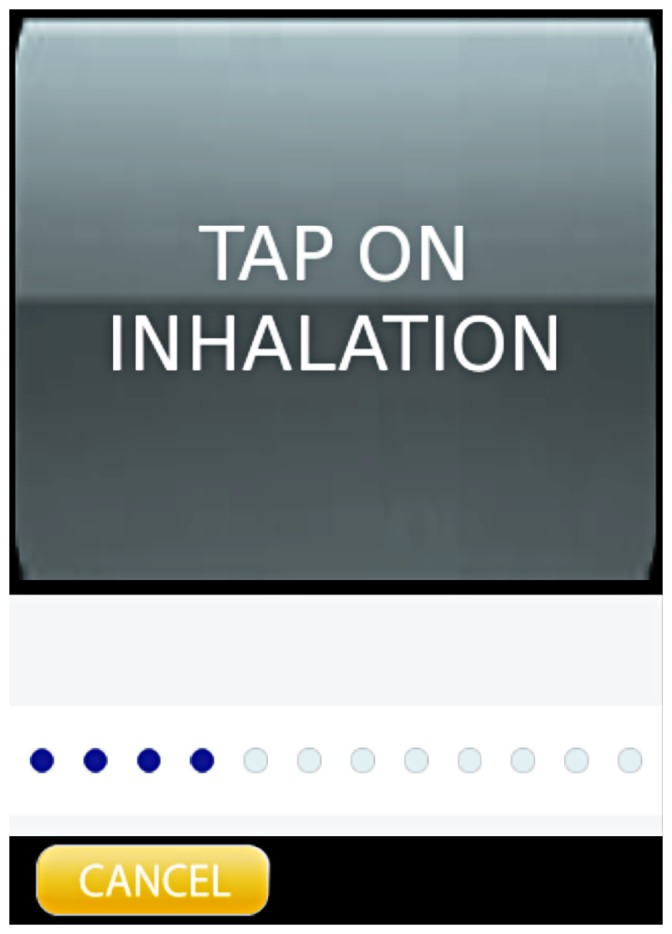
Screenshot of tapping screen of the *RRate* application. A button records taps and an indicator displays how many taps have been performed (bottom).

**Figure 3:**
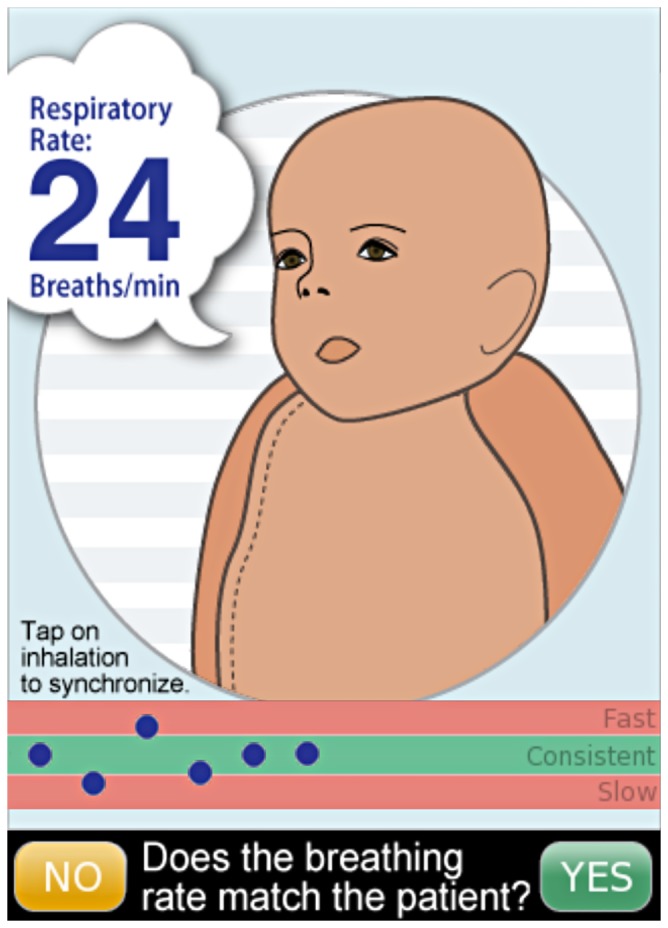
Screenshot of feedback screen of the *RRate* application. An animated (chest, shoulder and mouth) baby presents the RR. The timing of the animation can be reset with a tap. The consistency of the tap intervals is displayed on the bottom of the screen as blue dots.

The application allows the user to measure at any RR between 2 and 140 breaths/min and displays the number of taps completed (Figure 2). When a set of tap intervals with a consistency smaller or equal than 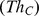 is obtained, a sound is played (chimes) and a second screen is presented to the user with an animated baby, breathing at the frequency of the calculated RR (Figure 3). The user can compare the breathing movement of the patient with that of the animated baby in order to confirm the accuracy of the calculated RR. The animation is supported through vibro-tactile feedback, reducing distractions caused by auditory feedback.

For conducting the present study, the *RRate* application was modified to accommodate all requirements specific to each study phase and then installed on an iPod Touch 3*^rd^* generation (Apple Inc, Cupertino, CA, USA) with a screen resolution of 480×320 pixels.

### Ethics statement

The protocol for this study (video recording and assessment of accuracy) was approved by The University of British Columbia/Childrens and Womens Health Centre of British Columbia Research Ethics Board, Vancouver, Canada (#H13-01116). Written informed consent was obtained from study subjects or in the case of minors, from their parent or guardian, before enrollment into the study.

### Standard videos

De-identified video recordings were made of 23 anesthetized children, aged 0–5 years, breathing for a period of 3–5 minutes, at the British Columbia Children's Hospital, Vancouver, Canada. Only the exposed chest and abdomen were included in the field of view of the video. No facial features or any other identifying features were recorded.

Five cases of controlled and 5 cases of spontaneous ventilation were selected for use as standard videos. The RR ranged from 17 to 59 breaths/min (Table 1), and 3 cases included fast breathing (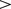
40 breaths/min) for the corresponding age [1]. The videos were each cropped to 60 s, the standard observation time for RR according to the WHO [7]. A still image of the first frame was played for 10 s at the start of each video to allow observers time to orient themselves to the position and anatomy of the child. Two independent observers (HG and MC) confirmed the RR of the selected videos by counting the number of breaths from the final videos by slowing the playback speed to 50% of the recorded speed. Each observer repeated the counting until the RR from three consecutive observations were in agreement.

**Table 1:** Standardized Videos.

Ventilation	Age (months)	RR (breaths/min)
Controlled	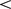 1	56
	23	33
	23	59*
	36	47*
	43	51*
Spontaneous	23	30
	23	38
	25	24
	53	17
	59	17

Ventilation mode, age and reference respiratory rate (RR) for the standard video recordings used in this study. The RRs labeled with an asterisk (*) are considered fast breathing for the corresponding age[1].

### Data collection

Adult subjects were recruited among trainee, volunteer and staff population from the British Columbia Children's Hospital. Their age, gender, education level, profession and mobile phone use familiarity [16] were recorded. For each subject, all 10 standard videos were played in a randomized order. The subjects measured RRs from the video recordings using the study specific version of the mobile phone *RRate* application. The study was conducted in two phases. In Phase I the subjects used the tapping application for an entire minute where the test for consistency was disabled (no Th*_C_*) in the *RRate* application. These data were used to perform a sensitivity analysis to understand the trade-off between accuracy and efficiency while changing the algorithm parameters Th*_C_* and z.

In Phase II the test for consistency was enabled to provide a more realistic use scenario. The parameters were set to a conservative threshold (z = 4, Th*_C_* = 6) derived from Phase I. The conservative thresholds were chosen to obtain a large dataset providing sufficient flexibility for conducting an optimization experiment to determine an ideal parameter configuration. Data obtained from Phase I and II are publicly available from the researchers' website (http://www.phoneoximeter.org/projects/rrate/).

### Sensitivity analysis

We performed a sensitivity analysis on Phase I data where 60 s of tapping was available to study the effects of Th*_C_* and the number of tap intervals z in a set, on the performance of the RR estimation. For performance, we were interested in accuracy (“How accurate is the RR estimation?”) and efficiency (“How fast can the result be obtained?”). The sensitivity analysis establishes the relationship between accuracy and efficiency. The sensitivity analysis was computed by varying z (the numbers of time intervals in a set) from 2 to 15 time intervals and varying Th*_C_* (the consistency thresholds) from 2% to 30%. This sensitivity analysis was then used to establish the settings of the application for Phase II.

#### Accuracy

The estimated RR was compared to the reference RR for accuracy. We used normalized root mean square error (NRMSE) as a measure for accuracy, such as
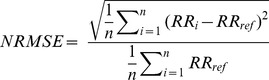
where n is the number of observations and RR*_ref_* is the reference RR obtained by expert observation. The NRMSE has the advantage of emphasizing the magnitude of the variance when the estimator is unbiased, penalizing large errors. This is of clinical importance where small errors are less relevant.

#### Efficiency

The time taken for completing the tapping measurement depends on the RR to be measured, the number of tap intervals z required in a set, and the consistency threshold Th*_C_*. A RR is only reported when the user has made consistent taps in all the time intervals of the latest set. Efficiency (E) measures the time from the first tap until the RR is reported such as
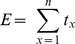



In cases where a valid set was not completed for a given combination of parameters within 60 s, E was set to 60 s, no RR was reported and the completion rate (CR) was reduced, thus penalizing the parameter choice. CR was calculated such as
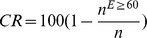
where 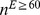 is the number of observations that did not complete within 60 s.

#### Relationship Modeling

We built a linear regression model to determine the relationship between NRMSE, E and the number of consecutive time intervals z and the consistency threshold Th*_C_*. We also conducted a model diagnostic based on residual plots to verify that the linear regression assumptions (i.e. random errors are normally distributed with mean = 0 and constant variance) were fulfilled. Details of this modeling can be found in the Materials S1.

### Optimization

There is a trade-off between accuracy and efficiency. While a small consistency threshold Th*_C_* can lead to high accuracy, it may be too restrictive and exclude natural breathing variations. A restrictive Th*_C_* increases the number of required taps to achieve a valid set or prevents the achievement of such a set in a reasonable time. Similarly, larger sets increase the accuracy, but also increase the minimum number of taps to complete the set. The optimal combination of z and Th*_C_*:

1. gives the most accurate RR measurement (lowest NRMSE);
2. in the least amount of time (in terms of median and 95*^th^* percentile time taken); and
3. with the highest CR (where a RR is reported in 60 s or less).

For this, we used a cost function analysis, such as
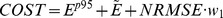
where 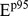 is the 95*^th^* percentile of the Efficiency, 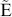 the median Efficiency and 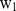 a weighting factor to balance out efficiency and accuracy. We considered the upper acceptable limit for 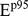 and 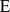 to be 15 s and 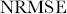 4%. Therefore the setting for 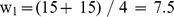 should provide an optimal balance with equal weighted error and time. The CR was taken into consideration by the fact that a failed measurement is penalized with E = 60.

The optimization experiment was performed using a 15-fold cross-validation on the combined data set from Phase I and II. Two observations for each video were randomly assigned to one of 15 data bins. A single data bin was then selected as a test set and the remaining bins are used as a training set. This was repeated until all data bins, and consequently each observation, were used once as a test set. The parameters with the smallest costs were selected in each repetition. The optimal parameters from each repetition were then ranked and the most frequent occurrence was reported and selected to calculate the performance of the test sets. The performance of the RR from the median tap intervals of the selected tap interval number without consistency test (z = 4, no Th*_C_*) was calculated on the test sets and compared against the results from the optimization using a pairwise t-test at 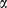 = 0.05 level.

## Results

### Subject demographics and recordings

Thirty-two adult subjects were recruited from the British Columbia Children's Hospital's trainee, volunteer and staff population. Twenty-two subjects measured RR using the mobile app for 60 s (Phase I). Ten subjects from the nursing staff measured RR using the mobile app until a consistent set was obtained (Phase II). Two subjects from Phase II were excluded for not completing all videos. Subject demographics and mobile phone use familiarity are summarized in Table 2. A total of 220 observations were obtained for Phase I and 80 for Phase II.

**Table 2:** Demographics.

		Phase I (60 s, no Th_*C*_)		Phase II (z = 4, Th_*C*_ = 6)	
Category	Subcategory	n	%	n	%
Age	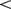 31 y	15	68.18	0	0.00
	31–40 y	5	22.73	1	12.50
	41–50 y	1	4.55	3	37.50
	51–60 y	1	4.55	4	50.00
Gender	Female	13	59.09	8	100.00
	Male	9	40.91	0	0.00
Highest Education	High School	4	18.18	0	0.00
	Undergraduate	10	45.45	4	50.00
	Postgraduate	8	36.36	4	50.00
Profession	Researcher	9	40.91	0	0.00
	Medical Student	7	31.82	0	0.00
	Engineer	6	27.27	0	0.00
	Registered Nurse	0	0	8	100.00
Type of Mobile User [16]	Voice/Text Fanatic	10	45.45	4	50.00
	Mobile Elite	5	22.73	1	12.50
	Minimalist	5	22.73	3	37.50
	Display Maven	2	9.09	0	0.00

Demographics and Mobile Phone Use Familiarity for subjects in Phase I (tapping for 60 s without applying a consistency test, no Th*_C_*) and Phase II (tapping until 4 tap intervals (z = 4) comply with Th*_C_* = 6).

### Sensitivity analysis

#### Influence of the number of time intervals on accuracy

**Figure 4:**
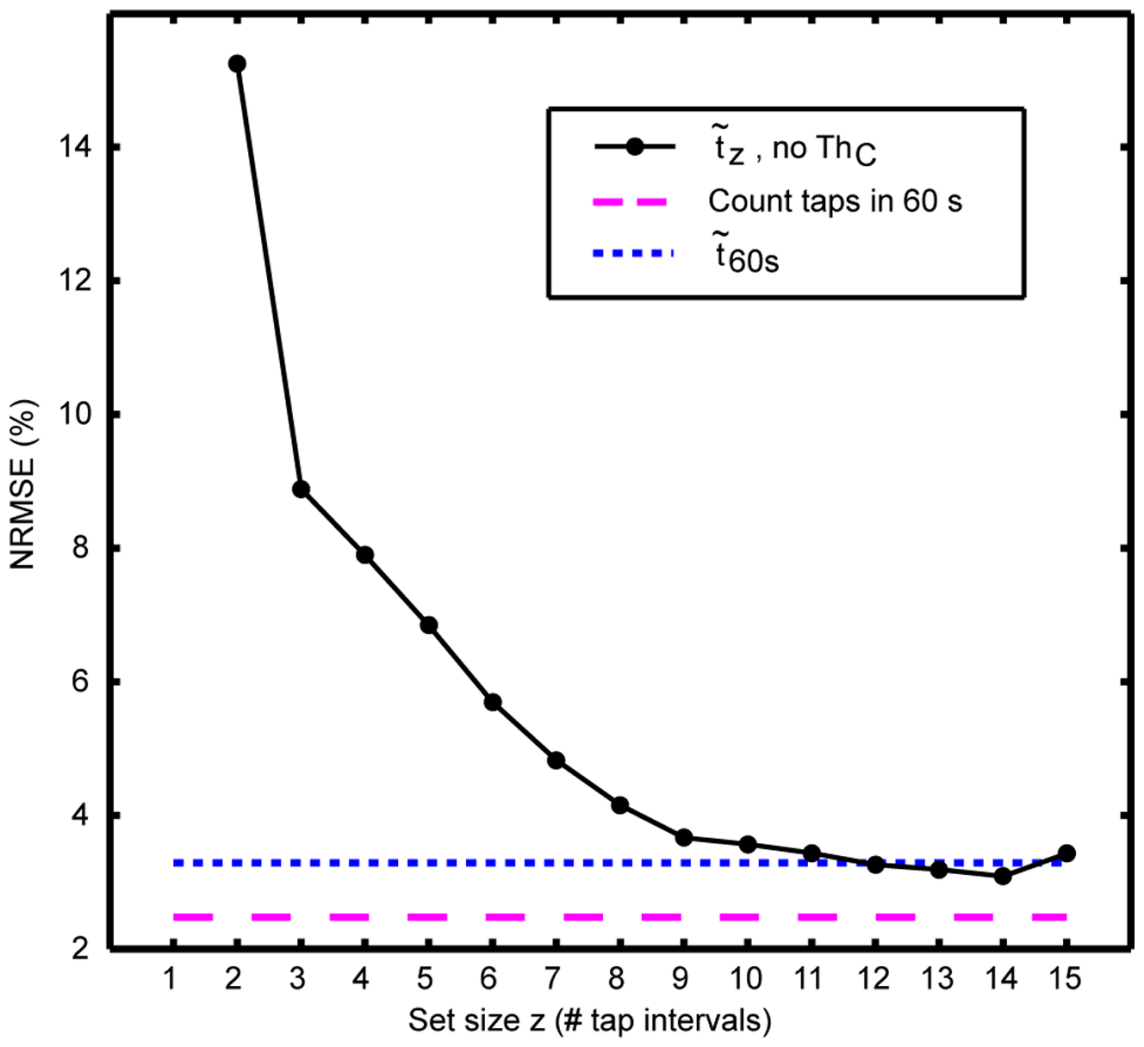
Improvement of RR estimation accuracy with larger set sizes z (continuous curve). The normalized root mean square error (NRMSE) of counting taps in 60 s is depicted as a dashed line; the NRMSE of the RR obtained from the median tap times of all taps in 60 s is depicted as a dotted line.

**Figure 5:**
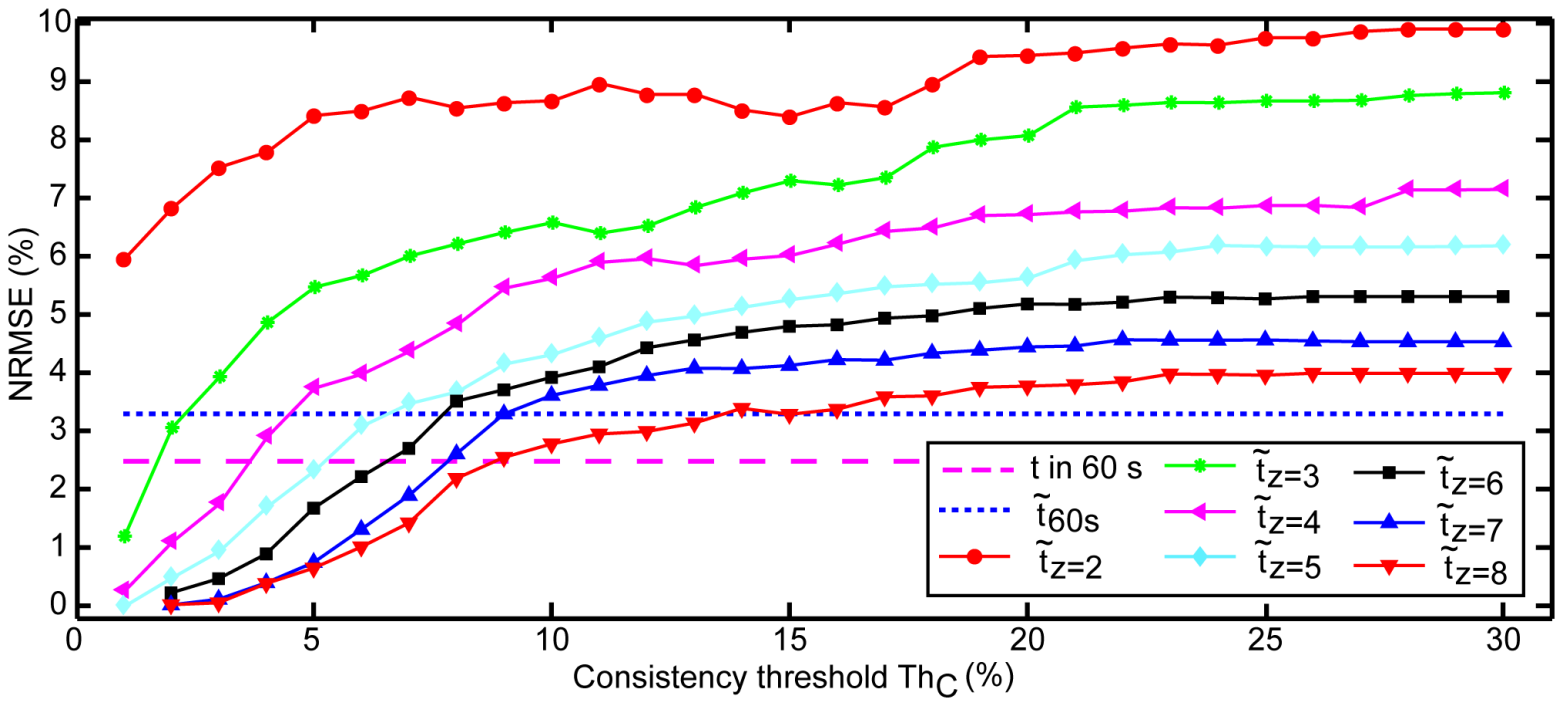
Plot of normalized root mean square error (NRMSE) against consistency threshold for different number of time intervals in a set. NRMSE decreased (accuracy improved) with tighter consistency thresholds and with increasing number of intervals in a set. At lower Th*_C_* and higher z there were fewer successfully completed cases (Figure 7) that contributed to the results.

There was a clear reduction of NRMSE with increased number of intervals (z) used to calculate the median respiratory interval, without using a Th*_C_* (Figure 4) and at different Th*_C_* (Figure 5). Without a Th*_C_*, the accuracy improved rapidly initially with increasing z, but trended towards NRMSE = 3.29% after z = 12, which was the accuracy that was obtained when using all available tap times in the 60 s experiment.

The linear regression model for NRMSE indicated that 97% of the variability in NRMSE can be accounted for by z and Th*_C_*. Therefore, we confirmed that the accuracy improves when the number of consecutive time intervals increases, provided that number did not exceed 12. We also concluded that for a fixed Th*_C_*, when z was no greater than 12, NRMSE significantly decreased, if z increased from x to x+1. Figures and statistical analysis for the models can be found in the Materials S2 and Materials S3.

#### Influence of consistency on accuracy

Accuracy improved (smaller NRMSE) when Th*_C_* was set tighter (Figure 5). From the linear regression model we confirmed that the accuracy improved when time intervals were more consistent since for a fixed z, NRMSE significantly decreased by 2.63–0.5y if Th*_C_* decreased from (y+1)^2^ to y^2^ for y = 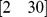.

#### Influence of consistency and the number of time intervals on efficiency

**Figure 6:**
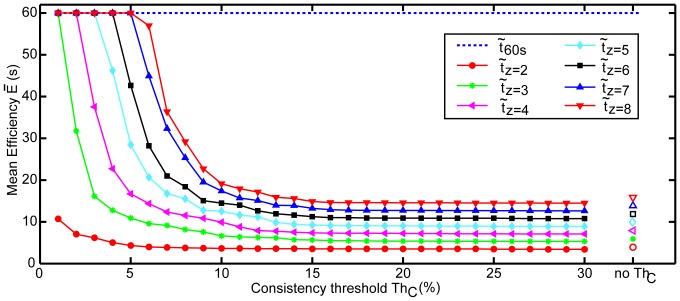
Plot of mean efficiency 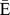 for measurement against consistency threshold Th*_C_* for the different number of time intervals in a set (z). 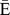
 became greater with tighter Th*_C_* and with increasing z.

**Figure 7:**
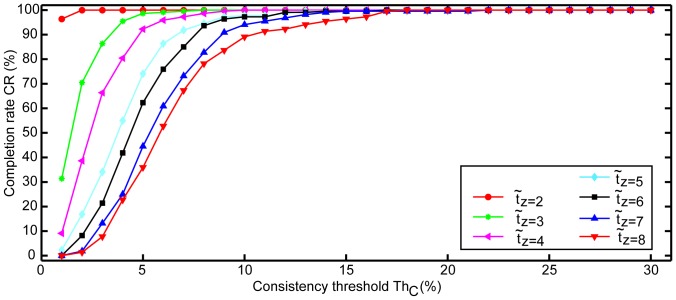
Completion rate CR against consistency threshold Th*_C_* for the different number of time intervals in a set (z).

The mean time taken for measurement increased when the number of time intervals in a set increased, and when Th*_C_* was set tighter (Figure 6). While accuracy improved with tighter parameters, time to completion increased until no estimation could be obtained within 60 s (Figure 7). The linear regression model confirmed that the median efficiency 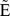 significantly decreased as z increased for a fixed Th*_C_* (p-value
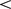
0.001), and also significantly increased as Th*_C_* increased for a fixed z (p-value
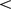
0.001). This also held true for the 95*^th^* percentile of the time taken to measure a RR. Thus we confirmed that the time taken to measure a RR depended on the number of time intervals and the consistency threshold.

### Optimization

**Figure 8:**
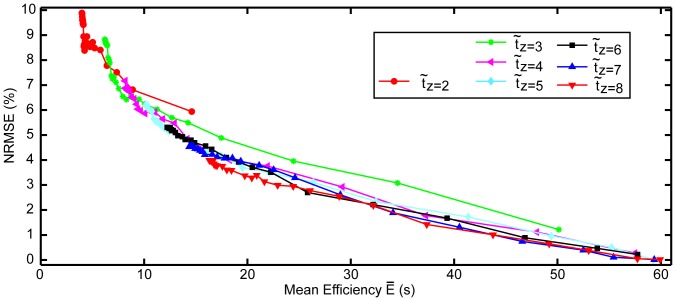
Plot of normalized root mean square error (NRMSE) against mean efficiency 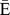 for different consistency thresholds Th*_C_* and different number of time intervals in a set (z). Increasing the accuracy (smaller NRMSE) is at the cost of increasing 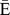.

Improving the accuracy (smaller NRMSE) was at the cost of increasing the time taken for measurement (Figure 8). Figure 8 also shows that z = 4 displayed a similar trend to set sizes z
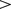
4, as opposed to z = 2 and z = 3. Set size z = 4 was consequently chosen for Phase II. For this set size, Th*_C_* = 6 was the most conservative threshold that would still allow a mean efficiency 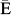 below 20 s (Figure 6) and a CR of 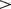
95% (Figure 7).

**Figure 9:**
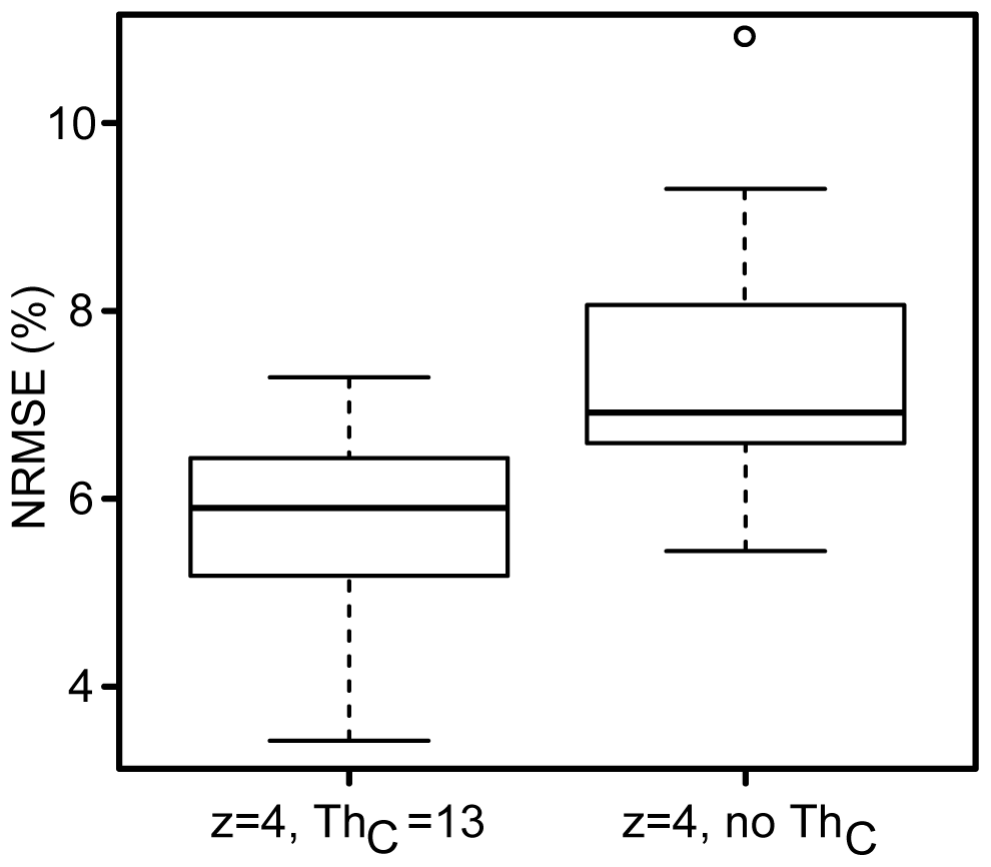
Distribution of normalized root mean square error (NRMSE) of the optimization results for experiments with consistency threshold (Th*_C_* = 13) and without. Adding a Th*_C_* improves the NRMSE significantly. The horizontal lines of each box are the lower quartile, median, and upper quartile values (from bottom to top). The whiskers represent the most extreme values within 1.5 times the interquartile range from the quartile. The outlier (circle) is a value beyond the interquartile range.

**Figure 10:**
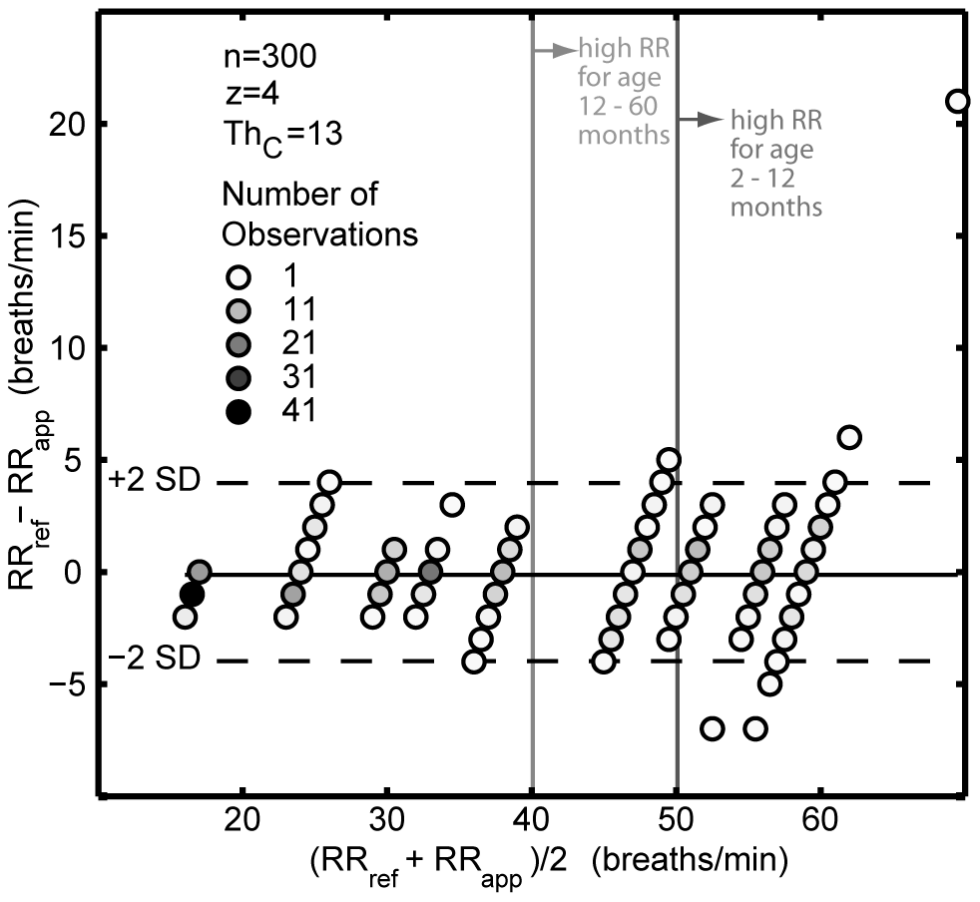
Bland-Altman plot of the optimal parameter configuration (z = 4, Th*_C_* = 13) for the RRate application, determined by data from Phase I and II (n = 300). The mean difference (bias) is −0.13 breaths/min and the standard deviation (SD) is 1.98 breaths/min. The number of observations is displayed as marker intensity. The dashed lines represent the 95*^th^* percentile range. The vertical lines correspond to the limits for fast breathing by age [1].

The cost function analysis revealed that the parameter combination z = 4, Th*_C_* = 13 ranked highest with lowest cost, being selected unanimously at each cross-validation repetition. For these 15 repetitions, the NRMSE was 5.6%±1.1 (Table 3). As shown previously with the modeling, this was significantly better (p = 0.018) than when using z = 4, no Th*_C_* (Figure 9). Bias between RR obtained from tapping and reference observation was −0.13 breaths/min (Figure 10). Only higher RRs (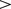 40 breaths/min) lead to observations with errors outside the 95*^th^* percentile (±2 SD) error range.

**Table 3:** Cross-Validation Results.

15 repetitions	z = 4, Th*_C_* = 13	z = 4, no Th*_C_*
NRMSE (%)	5.6±1.1	7.4±1.4
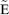 (s)	8.1±1.2	6.9±0.1
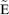 (s)	9.9±0.6	7.9±0.2
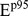 (s)	17.6±2.7	14.9±0.2
CR (%)	100	100

Comparison of normalized root mean square error (NRMSE), median efficiency (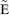), mean efficiency (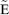), 95*^th^* percentile efficiency (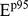) and completion rate (CR) for the optimal parameters z = 4, Th*_C_* = 13 and z = 4 without Th*_C_* (mean ± standard deviation).

## Discussion

We developed a mobile phone application that calculates the RR by measuring the time intervals between breaths by tapping the screen of a mobile device. Efficiency E was significantly improved compared to conventional methods that require counting for 60 s. The introduction of a consistency threshold Th*_C_* eliminated inconsistent user input and reduced the NRMSE significantly.

The mobile phone application *RRate* required the user to tap on a touch sensitive screen in time with inhalation, and the tap interval times were recorded into a set. The median of a set of tap intervals was calculated and each interval was checked for consistency. If all intervals were within a consistency range, the RR was reported as 60 s divided by the median. With our experiments, we have demonstrated that the median efficiency 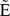 improved from 60 s to a mean of 8.1±1.2 s and a mean 95*^th^* percentile of 17.6±2.7 s. This was a significant reduction of time required for achieving a reasonable estimation of RR. The mean NRMSE of 5.6% corresponded to an error of 2.2 breaths/min at the critical RR of 40 breaths/min which is the diagnostic threshold for fast breathing in children aged 1 to 5 years [1].

Accuracy increased with the number of breaths included in a set of taps to calculate the median. When ignoring the Th*_C_* the reduction in error with more tap intervals was evident. This effect was most pronounced when increasing small tap intervals numbers, such as from z = 2 to z = 3. We also confirmed this finding when Th*_C_* was enabled using the regressive modeling.

The sensitivity analysis showed that excluding inconsistent tapping improved the estimation for a large range of tapping interval numbers. The measurement of consistency (maximum deviation of the median) allowed for instant rejection of aberrant taps. It also gave a measure of confidence to the performed measurement. The consistency between the taps performed depended on the natural variation of breathing and the accuracy of the taps performed by the user. An excessively restrictive consistency threshold would exclude natural variation of breaths and impact the usability of the method, forcing the user to tap for longer than 60 s. On the other hand, an overly relaxed consistency threshold would allow for user mistakes and negatively impact accuracy. Similarly, including a large number of breaths in a set would be detrimental to efficiency by increasing the minimum number of times a user had to tap before obtaining a RR, while using very few breaths to calculate the median would decrease accuracy. In previous work we have shown that accuracy of RR estimations obtained simultaneously from three independent sources can be improved when testing for agreement and excluding observations with large deviations from the mean RR [17]. The algorithm presented in the present manuscript successfully applied similar principles to consecutive observations from a single source.

The standard videos used for the sensitivity analysis contained a wide spectrum of RRs, including fast breathing. However, these videos do not represent all possible breathing patterns. For example, in neonates the breathing variation is increased through periodic breathing, a normal breathing pattern characterized by alternating between regular breathing and short periods of apnea [18]. In such groups of patients, the estimated RR may not match the actual RR. A Th*_C_* not customized to this patient group may also be too restrictive and increase the required assessment time. Also, only a relative small number of health workers (32) have tested the *RRate* application in a laboratory setting for this initial evaluation. However, each subject did perform 10 independent patient observations. Further tests with a higher number of health workers and patients with a broader range of breathing patterns are required to generalize our findings to a broader group of patients. Before the release of the application, a larger study will be required to test robustness of the proposed method in the target setting.

The improvement in estimation of a rate using the continuous analysis of time intervals instead of counting of events in a fixed time interval is not entirely new and has been shown to be effective in improving efficiency and accuracy of rate estimations [10], [11]. In [10] it was highlighted that the trade-off between accuracy and efficiency is a design choice. We showed that such a trade-off between accuracy and efficiency is present for RR estimation and provided a cost model for optimizing this trade-off. We have used a generic cost function that offers a balance between efficiency and accuracy. Depending on specifications of an application, e.g. type of diagnosis or measurement setting, the cost function can be modified to obtain the optimal parameters for the desired task. Understanding the relationship between accuracy and efficiency is crucial when designing new RR counters. The proposed methodology is an essential step for facilitating device specification developments and a useful tool to define a target product profile for RR estimation devices for resource limited settings. Usability studies that will evaluate user acceptance of measurement time and other design aspects will have to accompany the development of device specification for these settings.

Mobile technology is ubiquitous, even in developing countries and rural parts of the world [12]. A growing number of initiatives are using mobile phones to tackle the health challenges of developing countries [12]. One of these challenges is the accurate measurement of RR in children. We proposed a RR estimation algorithm based on measuring the time intervals between breaths that allows a 6-fold increase in mean efficiency compared to the current recommended method of counting breaths for 60 s with the ARI Timer. This algorithm was implemented into the *RRate* mobile application, which is freely available for download [14], [15]. With the reduction in cost and widespread use of smart phones and mobile devices, such mobile applications may be a promising replacement for the ARI Timer. Vibro-tactile and visual feedback allows the user to focus on the patient and to obtain feedback for quality assurance at completion of measurement. We are currently undertaking a direct comparison between the *RRate* and the ARI Timer for accuracy, efficiency and usability. Further validation including clinical testing in the target environment with community health workers assessing sick children will be necessary to demonstrate positive impact on diagnosis of pneumonia and other respiratory diseases with the *RRate* application.

## Supporting Information

Materials S1 **Linear Modeling of Efficiency and NRMSE.**
(PDF)

Materials S2 **Regression Model of NRMSE.**
(PDF)

Materials S3 **Regression Model of Median Efficiency.**
(PDF)
