# Correction: Improving the Accuracy and Efficiency of Respiratory Rate Measurements in Children Using Mobile Devices

**DOI:** 10.1371/journal.pone.0118260

**Published:** 2015-02-06

**Authors:** The PLOS ONE Staff

There are errors in Table 3 that were introduced during the typesetting process. Please view the corrected Table 3 here. The publisher apologizes for these errors.

**Table 3:** Cross-Validation Results.

15 repetitions	z = 4, Th_C_ = 13	z = 4, no Th_C_
NRMSE (%)	5.6 ± 1.1	7.4 ± 1.4
$\tilde{E}$ (s)	8.1 ± 1.2	6.9 ± 0.1
$\overset{-}{E}$ (s)	9.9 ± 0.6	7.9 ± 0.2
E^p95^ (s)	17.6 ± 2.7	14.9 ± 0.2
CR (%)	100	100

Comparison of normalized root mean square error (NRMSE), median efficiency ($\tilde{E}$), mean efficiency ($\overset{-}{E}$), 95^th^ percentile efficiency (E^p95^) and completion rate (CR) for the optimal parameters z = 4 Th_C_ = 13 and z = 4 without Th_C_ (mean ± standard deviation).

## References

[1] Karlen W, Gan H, Chiu M, Dunsmuir D, Zhou G, et al (2014) Improving the Accuracy and Efficiency of Respiratory Rate Measurements in Children Using Mobile Devices. PLoS ONE 9(6): e99266 doi: 10.1371/journal.pone.0099266 24919062 10.1371/journal.pone.0099266 PMC4053345

