# Supplementary material for: Improving the Accuracy and Efficiency of Respiratory Rate Measurements in Children Using Mobile Devices
Source: PLoS One. 2014 Jun 11;9(6):e99266. doi: 10.1371/journal.pone.0099266 (PMC4053345; doi:10.1371/journal.pone.0099266)
Supplement: Materials S1 — Linear Modeling of Efficiency and NRMSE. (PDF) [file pone.0099266.s001.pdf]

S1 Linear Modeling of Efficiency and NRMSE

We built a linear regression model to check the relationship between NRMSE, E and the number of consecutive time intervals z and the consistency threshold Th_C. The models have the form

$$\widehat{NRMSE} = a_1 + a_2 z + a_3 z^2 + a_4\sqrt{Th_C} + a_5 Th_C + err$$

and

$$\sqrt{\hat{E}} = b_1 + b_2 z + b_3 z^2 + b_4 e^{-0.15 Th_C} + err$$

Where err is a random error and a_i and b_i are the polynomial parameters of the model. The addition of $\sqrt{Th_C}$ and $e^{-0.15Th_C}$ was to ensure that the linear regression assumptions were approximately satisfied. We also conducted a model diagnostic based on residual plots to verify that the linear regression assumptions (i.e. random errors are normally distributed with mean 0 and constant variance) were fulfilled.
