# Supplementary material for: Improving the Accuracy and Efficiency of Respiratory Rate Measurements in Children Using Mobile Devices
Source: PLoS One. 2014 Jun 11;9(6):e99266. doi: 10.1371/journal.pone.0099266 (PMC4053345; doi:10.1371/journal.pone.0099266)
Supplement: Materials S2 — Regression Model of NRMSE. (PDF) [file pone.0099266.s002.pdf]

S2 Regression Model of NRMSE

All coefficients of the regression model for NRMSE were highly significant (p-values are all <0.001, table S1). The model is fairly accurate with an Rˆ2 value 0.97, indicating that 97% of the variability in NRMSE can be accounted for by z and Th$_C$. Therefore, we confirm that the accuracy improves when the number of consecutive time intervals used increases, provided that number does not exceed 12 (Figure S1).

The regression formula is

$$\widehat{NRMSE} = 4.11 - 1.50z + 0.06z^2 + 2.88\sqrt{Th_C} - 0.25Th_C + err$$

Thus, for a fixed Th$_C$, when z is no greater than 12 and increases from x to x+1, the NRMSE is expected to decrease by

$$4.11 - 1.50x + 0.06x^2 - [4.11 - 1.50(x+1) + 0.06(x+1)^2] = 1.44 - 0.12x.$$

This decrease quantity is non-negative when $n \leq 12$; that is, the change in the expected NRMSE (i.e. the trend) is going to reverse direction when n exceeds 12. For example, NRMSE decreases by 1.2 when z increases from z=2 to z=3, and the decrease is 1.06 if z increases from z=3 to z=4.

For a fixed z, the expected decease in NRMSE when Th$_C$ decreases from $(y+1)^2$ to y^2 is

$$2.88\sqrt{(y+1)^2} - 0.25(y+1)^2 - [2.88\sqrt{y^2} - 0.25y] = 2.63 - 0.50y.$$

Therefore, we confirm that the accuracy improves when time intervals are more consistent.

For example, NRMSE decreases by 1.63 when Th$_C$ decreases from 9% to 4%, and it deceases by 2.13 when Th$_C$ from 4% to 1%.

The quantity $2.63 - 0.50k$ is negative when $k >= 5.26$. So the decreases in NRMSE when Th$_C$ decreases from $(6+1)^2 = 49$ (%) is to $6^2 = 36$ (%) is negative according to the model. This is because the input value is beyond the range of the data on which the model was built, which is 30% in our case.

Table S1. NRMSE Model Coefficients

Coefficient	Estimate	Std. Error	t value	Pr($>|t|$)
a_1	4.1054	0.2321	17.7	<0.001 ***
a_2	-1.4973	0.0278	-54.0	<0.001 ***
a_3	0.0587	0.0016	36.6	<0.001 ***
a_4	2.8828	0.1271	22.7	<0.001 ***
a_5	-0.2498	0.0175	-14.3	<0.001 ***

Coefficients and significance for NRMSE linear model.

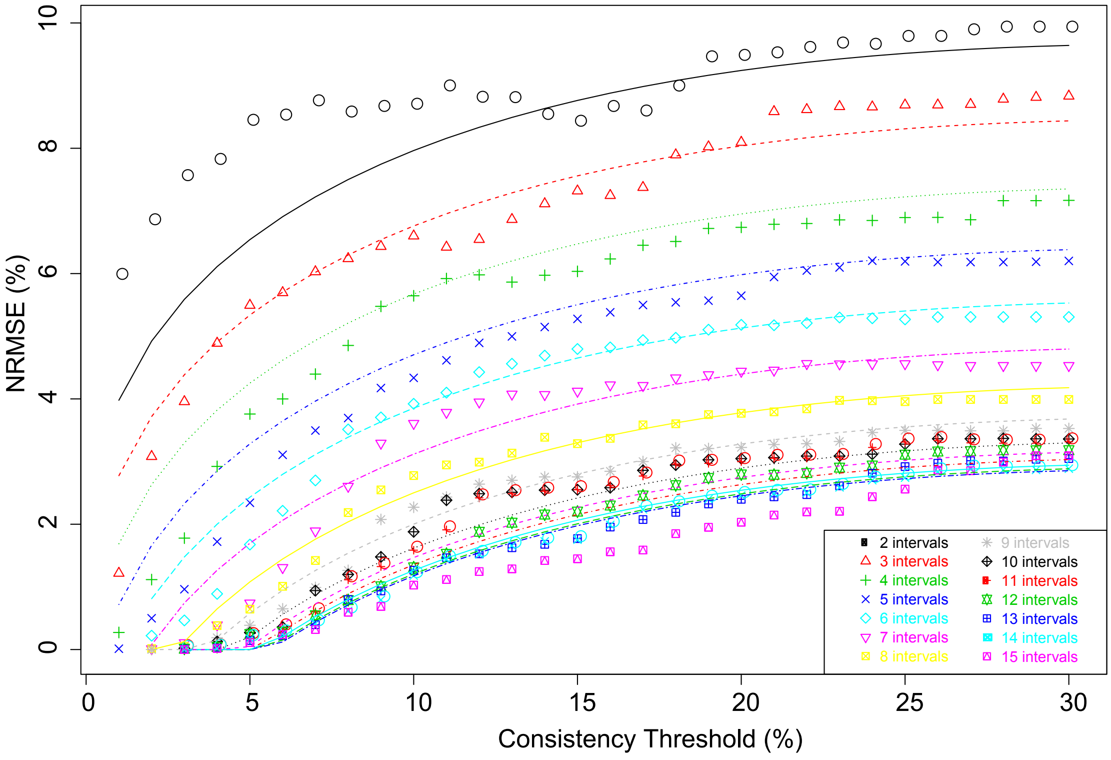

Figure S1. Linear model of the consistency/NRMSE relationship.
