# Supplementary material for: Improving the Accuracy and Efficiency of Respiratory Rate Measurements in Children Using Mobile Devices
Source: PLoS One. 2014 Jun 11;9(6):e99266. doi: 10.1371/journal.pone.0099266 (PMC4053345; doi:10.1371/journal.pone.0099266)
Supplement: Materials S3 — Regression Model of Median Efficiency. (PDF) [file pone.0099266.s003.pdf]

S3 Regression Model of median Efficiency

The coefficients b_2-b_4 of the regression model for $\tilde{E}$ were significant (p-values < 0.001, table S2). The model is accurate with an R^2 value 0.87, indicating that 87% of the variability in $\tilde{E}$ can be accounted for by z and Th_C. Therefore, we confirm that the efficiency improves when the number of consecutive time intervals used increases, provided that number does not exceed 12 (Figure S2).

Table S2. Efficiency Model Coefficients

Coefficient	Estimate	Std. Error	t value	$Pr(> \|t\|)$
b_1	0.20867	0.12410	1.68	0.094
b_2	0.62839	0.03871	16.23	<0.001 ***
b_3	-0.02219	0.00274	-8.09	<0.001 ***
b_4	3.81354	0.16713	22.82	<0.001 ***

Coefficients and significance for the efficiency E linear model.

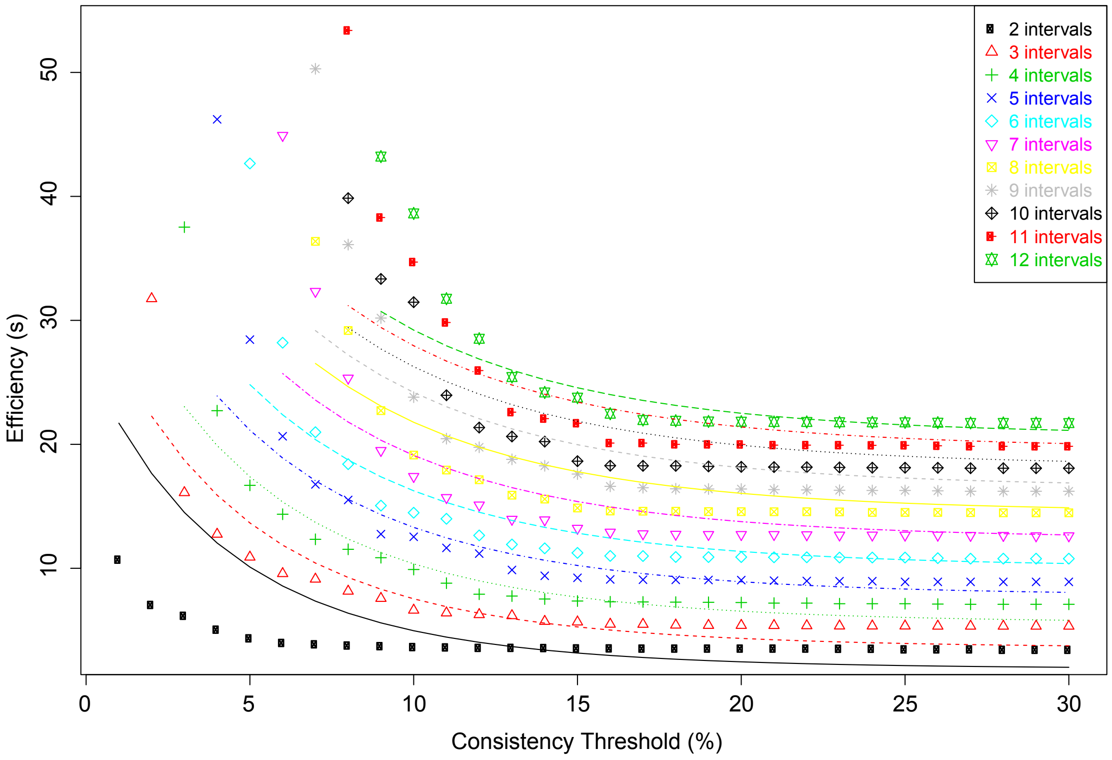

Figure S2. Linear model of the consistency/efficiency relationship.
